# BRAZILIAN GASTRIC CANCER ASSOCIATION GUIDELINES (PART 1): AN UPDATE ON DIAGNOSIS, STAGING, ENDOSCOPIC TREATMENT AND FOLLOW-UP

**DOI:** 10.1590/0102-672020200003e1535

**Published:** 2020-12-18

**Authors:** Leandro Cardoso BARCHI, Marcus Fernando Kodama Pertille RAMOS, Osmar Kenji YAGI, Donato Roberto MUCERINO, Claudio José Caldas BRESCIANI, Ulysses RIBEIRO, Nelson Adami ANDREOLLO, Paulo Pimentel ASSUMPÇÃO, Antônio Carlos WESTON, Ramiro COLLEONI, Bruno ZILBERSTEIN, Consensus members, Álvaro Antônio Bandeira Ferraz, Amir Zeide Charruf, André Roncon Dias, André Brandalise, André Maciel da Silva, Barlon Alves, Carlos Alberto Malheiros, Carlos Augusto Martinez Marins, Celso Vieira Leite, Daniel Szor, Durval R. Wohnrath, Elias Jirjoss Ilias, Euclides Dias Martins, Fabio Pinatel Lopasso, Felipe José Fernandez Coimbra, Fernando E. Cruz Felippe, Flávio Daniel Saavedra Tomasisch, Flavio Roberto Takeda, Geraldo Ishak, Gustavo Andreazza Laporte, Herbeth José Toledo Silva, Ivan Cecconello, Joaquim José Gama Rodrigues, José Carlos Del Grande, Laércio Gomes Lourenço, Leonardo Milhomem da Motta, Leonardo Rocha Ferraz, Luis Fernando Moreira, Luis Roberto Lopes, Marcelo Garcia Toneto, Marcelo Mester, Marco Antônio Gonçalves Rodrigues, Marineide Prudêncio de Carvalho, Maurice Youssef Franciss, Nora Manoukian Forones, Oly Campos Corletta, Osvaldo Antonio Prado Castro, Osvaldo Malafaia, Paulo Kassab, Paulo Roberto Savassi-Rocha, Rodrigo Jose de Oliveira, Rubens Antonio Aissar Sallun, Rui Weschenfelder, Saint Clair Vieira de Oliveira, Thiago Boechat de Abreu, Tiago Biachi de Castria, Williams Barra, Wilson Luiz da Costa, Wilson Rodrigues de Freitas

**Affiliations:** 1 Hospital das Clinicas HCFMUSP, Faculty of Medicine, University of São Paulo, São Paulo, SP, Brazil; 2 Faculty of Medicine São Leopoldo Mandic, Campinas, SP, Brazil; 3 State University of Campinas, Campinas, SP, Brazil; 4 Federal University of Pará, Núcleo de Pesquisas em Oncologia; 5 Department of Surgery, Santa Casa de Porto Alegre, Porto Alegre, RS, Brazil; 6 Department of Surgery, School of Medicine, Federal University of São Paulo, São Paulo, SP, Brazil

**Keywords:** Gastric cancer, Guidelines, Staging, Endoscopic treatment, Consensus, Follow-up, Câncer gástrico, Diretriz, Estadiamento, Tratamento endoscópico, Consenso, Seguimento

## Abstract

**Background:**

The II Brazilian Consensus on Gastric Cancer by the Brazilian Gastric Cancer Association (ABCG) was recently published. On this occasion, several experts in gastric cancer expressed their opinion before the statements presented.

**Aim:**

To present the ABCG Guidelines (part 1) regarding the diagnosis, staging, endoscopic treatment and follow-up of gastric cancer patients.

**Methods:**

To forge these Guidelines, the authors carried out an extensive and current review regarding each statement present in the II Consensus, using the Medline/PubMed, Cochrane Library and SciELO databases with the following descriptors: gastric cancer, staging, endoscopic treatment and follow-up. In addition, each statement was classified according to the level of evidence and degree of recommendation.

**Results:**

Of the 24 statements, two (8.3%) were classified with level of evidence A, 11 (45.8%) with B and 11 (45.8%) with C. As for the degree of recommendation, six (25%) statements obtained grade of recommendation 1, nine (37.5%) recommendation 2a, six (25%) 2b and three (12.5%) grade 3.

**Conclusion:**

The guidelines presented here are intended to assist professionals working in the fight against gastric cancer with relevant and current information, granting them to be applied in the daily medical practice.

## RESUMO

**Racional:**

O II Consenso Brasileiro de Câncer Gástrico da Associação Brasileira de Câncer Gástrico (ABCG) foi recentemente publicado. Nesta ocasião, inúmeros especialistas que atuam no tratamento desta doença expressaram sua opinião diante declarações apresentadas.

**Objetivo:**

Apresentar as Diretrizes da ABCG (Parte 1) quanto ao diagnóstico, estadiamento, tratamento endoscópico e seguimento dos pacientes com câncer gástrico.

**Métodos:**

Para formulação destas Diretrizes os autores realizaram extensa e atual revisão referente a cada declaração presente no II Consenso, utilizando as bases Medline/PubMed, Cochrane Library e SciELO com os seguintes descritores: câncer gástrico, estadiamento, tratamento endoscópico e seguimento. Ainda, cada declaração foi classificada de acordo com o nível de evidência e grau de recomendação.

**Resultados:**

Das 24 declarações, duas (8,3%) foram classificadas com nível de evidência A, 11 (45,8%) B e 11 (45,8%) C. Quanto ao grau de recomendação, seis (25%) declarações obtiveram grau de recomendação 1, nove (37,5%) grau 2a, seis (25%) 2b e três (12,5%) 3.

**Conclusão:**

As diretrizes aqui presentes têm a finalidade de auxiliar os profissionais que atuam no combate ao câncer gástrico com informações relevantes e atuais, permitindo que sejam aplicadas na prática médica diária.

## INTRODUCTION

Gastric cancer (GC) is a complex disease, with different forms of presentation and clinical evolution. Thus, many aspects related to diagnosis, staging, treatment and follow-up remain constantly changing. The Japanese Gastric Cancer Association recently published the 5^th^ English edition of the Japanese Gastric Cancer Treatment Guidelines. In this new edition, some evidence that recently emerged were presented, for instance: the new TNM staging classification of the Union for International Cancer Control (UICC) - 8^th^ edition; the new classification of curability after endoscopic resection, the abandonment of lymph node dissection of the splenic hilus (station nº 10) in D2 lymphadenectomy in total gastrectomy; among others^2^. Furthermore, the Brazilian Gastric Cancer Association (ABCG) recently published the II Brazilian Consensus on Gastric Cancer^7^. In this study, 67 statements regarding the diagnosis, staging, treatment and prognosis of GC were presented to 57 specialists from all regions of the country. The experts were carefully selected considering the notorious knowledge and contribution of each one in the GC field. In this opportunity, the participants responded to the statements with only one possible answer among the alternatives “I totally agree”; “Partially agree”; “Undecided”; “I disagree”; “Strongly disagree”. A consensus was adopted when at least 80% of the sum of the answers “fully agree” and “I partially agree” was reached. The results were presented at an ABCG event in Porto Alegre (RS).

As mentioned in the II Consensus, Brazil is a country with a continental dimension, with many regional peculiarities that directly impact on the diagnosis, treatment and prognosis of GC patients. Therefore, it is pertinent that each region can act in the fight against GC in the best possible way, according to the local reality. Therefore, the aim of this study is to interpret the statements regarding the diagnosis, staging, endoscopic treatment and follow-up contained in the II Brazilian Consensus on Gastric Cancer through a review of the most recent medical literature. Again, it is important to note that the comments contained here are not absolute whatsoever. The idea is to provide new concepts and update old knowledge, which will certainly benefit GC patients. That said, each information presented here must be carefully analyzed and used in the best possible way according to the resources available at your place of work.

## METHODS

During the elaboration of the methodology used in the II Consensus, some alternatives were presented in relation to the possibilities of the experts’ responses to the statements displayed: the method used in the I Consensus published in 2013 could be repeated, in which there were only two possible answers ( “yes or no”); or else, offer the opportunity to the experts to agree wholly or in part, as well as disagree wholly or in part with the statements presented. Both options have already been used in the literature^43^ ^,^ ^44^. The second option was chosen, since it can provide a greater amount of relevant information related to GC, when performing the comments presented in this study. In addition, as will be discussed below, there are many possibilities for managing GC, according to the situation in each region. 

To compose the comments for each statement, the authors used the Medline/PubMed, Cochrane Library and SciELO databases with the following descriptors: gastric cancer, staging, endoscopic treatment and follow-up. Preference was given to more recent articles with better statistical relevance. In addition, each statement was classified according to the level of evidence and degree of recommendation adapted from the Brazilian Medical Association/Federal Council of Medicine (AMB/CFM) Guidelines represented in Table 1 ^31^. Note that, in this first part, the ABCG Guidelines will only address issues related to diagnosis, staging, endoscopic treatment and follow-up. The ABCG Guidelines related to treatment itself will be addressed in a future publication. Note that the numbering of the statements is not in sequential order. They were divided according to the topic in question. 

**TABLE 1:** The correspondence between the degree of recommendation and the strength of scientific evidence

Levels of evidence:
Level A: when there are several randomized clinical trials supporting the evidence and consequently the recommendation
Level B: when there is only one randomized clinical trial, or non-randomized studies
Level C: when the evidence is based only on retrospective studies or expert opinion
Degrees of recommendation:
Class I: there is evidence and / or general agreement that a particular procedure is useful and effective
Class IIa: the evidence shows some conflicts, so there is a difference of opinion on the usefulness and effectiveness of the procedure (despite the divergence, the weight of opinion leans in favor of the usefulness / effectiveness of the procedure)
Class IIb: the evidence shows some conflicts, so there is a difference of opinion on the usefulness and effectiveness of the procedure (this utility / effectiveness is less well established)
Class III: there is no evidence in favor of a certain procedure

Adapted from the Guidelines of the Brazilian Medical Association / Federal Council of Medicine (AMB / CFM)31

## RESULTS

Of the 24 statements presented in this study, two (8.3%) were classified with level of evidence A, 11 (45.8%) with level B and 11 (45.8%) with level C. As for the degree of recommendation, six (25%) statements obtained grade of recommendation 1, nine (37.5%) 2a, six (25%) 2b and three (12.5%) 3.

### Diagnosis statements

#### Statement 1

The main method of gastric cancer diagnosis is through an upper gastrointestinal endoscopy with biopsy. The endoscopic examination report must contain precise information regarding location (s) of the lesion (s), approximate size, extent, infiltration, distance from the esophageal-gastric transition and the pylorus, detailing the places where the biopsies were performed. 100% Agreement (level of evidence B; degree of recommendation 1).

#### Comment

Hamashima *et. al.* (2018) reviewed the role of upper endoscopy and biopsies in GC. Among the 1,170 publications found between 2000 and 2013, 21 well-designed researches that use this screening method stood out. The authors emphasize that the endoscopic method is more sensitive in detecting GC, especially early GC, compared to radiological methods^17^. In addition, some case-control studies have demonstrated reduced GC mortality by endoscopic screening^20^. Finally, the latest version of the Japanese Gastric Cancer Treatment Guidelines emphasizes that *H. pylori* and serum pepsinogen antibody screening tests are not recommended as population screening methods due to the lack of scientific evidence^17^.

#### Statement 2

In case of high suspicion of gastric cancer and repeatedly negative biopsies collected by upper gastrointestinal endoscopy (including macrobiopsies), the diagnosis can be made through endoscopic resection or surgery. 94% Agreement (level of evidence C; degree of recommendation 2a)

#### Comment

Gastric biopsies must follow the Sydney Protocol (biopsy of the antrum, incisura angularis, small and greater curvature). It generally allows the diagnostic confirmation and the risk assessment for disease development. Anyhow, it is noteworthy that endoscopic mucosal resection or endoscopic submucosal dissection in suspected gastric lesions with dysplasia or early cancer are effective, with a high success rate and low recurrence. Another recommendation is enhanced image upper endoscopy and use of dyes, combined with biopsies is the best approach to accurately detect and stratify GC. Still, in cases where there is a high suspicion of GC, with repeated negative biopsies and highly suspicious imaging exams for GC, diagnostic laparoscopy can be performed^6^.

#### Statement 3

Ultrasound upper endoscopy is not indicated when there are clear endoscopic signs that the cancer is invasive. It should be used when there is any doubt about the early aspect of GC. It allows to evaluate the degree of tumor invasion in the gastric wall and the presence of suspicious lymph nodes for metastases. 96% Agreement (level of evidence C; degree of recommendation 2a)

#### Comment

According to a study published by Zaanan *et al.* (2018), the ultrasound upper endoscopy is indicated in the following situations: diffuse GC with negative biopsies; to determine the proximal and distal limits of the tumor; for lymph nodes diagnosis; to assess the extent of the lesion and when there is an indication for neoadjuvant chemotherapy, as the method allows lymph node biopsies and ascites analysis (which positivity can modified the strategy adopted). In addition, they conclude that ultrasound upper endoscopy is not useful in T3/T4 tumors diagnosed by computed tomography (CT)^41^.

Merkow *et al.* (2017) analyzed 734 patients treated for GC to assess the agreement between ultrasound upper endoscopy and the pathological result. The agreement was considered moderate (stage T: 52% and stage N: 70%). The accurately estimated risk of invasion was 73%, overestimated in 19% and underestimated in 8%. It may be concluded with this report that ultrasound upper endoscopy should be used with caution and when necessary, always associated with another diagnostic imaging method^27^.

#### Statement 4

The main staging method is computed tomography of the chest, abdomen and pelvis. 100% Agreement (level of evidence B; degree of recommendation 1)

#### Comment

Luo *et al.* (2017) published a meta-analysis to assess the value of CT in detecting lymph node metastases in GC. There were 27 studies enrolled, with 6,519 patients. They concluded that CT is adequate to assess lymph node metastases in the preoperative stage of serosa positive advanced GC. Nonetheless, it is insufficient to assess serosa negative GC (particularly early GC) due to its low sensitivity^25^. Another meta-analysis corroborated these results, showing that CT is more sensitive than ultrasound and ultrasound upper endoscopy. Another advantage of CT is the possibility of detecting distant metastases (mainly liver and lung) and the possibility to diagnosis peritoneal carcinomatosis^45^.

#### Statement 5

Positron emission tomography (PET-CT) and nuclear magnetic resonance (MRI) should be used only in selected cases. 100% Agreement (level of evidence C; degree of recommendation 2b) 

#### Comment

Some studies have shown good results with the use of MRI (positive predictive values, negative predictive values, sensitivities and specificities ranging from 67 to 100%, 71 to 100%, 50 to 100% and 63-100%, respectively). Possibly, MRI would have an added value in detecting lymph node metastases and systemic diseases, in defining the volume of tumors and in predicting treatment response. Still, there is the fact that there is no ionizing radiation emission and yet the benefit for patients allergic to iodinated contrast^10^ ^,^ ^14^. Kitajima *et al*. (2017) analyzed the use of PET-CT in GC staging and demonstrated its limited role in T stage evaluation. Considering its low level of spatial resolution, it provides limited information regarding gastric wall involvement or adjacent organs invasion. It is a method with lower sensitivity than isolated CT in detecting peritoneal lesions. Its sensitivity, specificity and precision for detecting lymph node metastases (N stage) varies from 41 to 74%, 75 to 100% and 51 to 76%, respectively. Whereas contrast CT scans vary from 70 to 83%, 62 to 92% and 67 to 80%, respectively. Possibly, the addition of PET-CT may assist in distant lymph nodes and bone metastases detection, which could significantly influence the treatment. After all, its use should be restricted to selected cases^22^.

#### Statement 6

PET-CT can be used in well-differentiated tumors or in the proximal third. 74% Agreement (level of evidence C; degree of recommendation 2b)

#### Comment

Gauthé *et al.* (2015) evaluated the application of PET-CT in GC staging and found that it is associated with a low detection rate (around 55%), especially in early GC, as well as signet ring cells, mucinous and poorly differentiated adenocarcinomas, which are typically less metabolically active. Well-differentiated tumors may eventually present greater uptake of the radiopharmaceutical fluorodeoxyglucose (FDG-^18^F) when compared to undifferentiated tumors. Notwithstanding, the variable and occasionally intense physiological uptake of FDG in the gastric wall is not uncommon and may mask the uptake by the primary tumor. Contrast uptake can often correspond to gastritis. As for proximal tumors, there is a benefit of using PET-CT in cardia tumors (mainly in Siewert’s type I and II adenocarcinomas). These tumors have different metabolic and biological behavior from true stomach cancers. In fact, they behave more similarly to esophageal tumors and so, PET-CT can assist in lymph node metastases detection in the chest, which often occur in these types of tumors^15^.

#### Statement 7

Staging laparoscopy should be performed in cases where there is uncertainty in computed tomography regarding the presence of peritoneal carcinomatosis or when multidisciplinary treatment is planned. 98% Agreement (level of evidence C; degree of recommendation 2a)

#### Comment

The staging laparoscopy with peritoneal washing cytology is recommended when CT fails to rule out the possibility of peritoneal carcinomatosis or radiologically occult metastatic disease. Recently, Li *et al.* (2020) conducted a study comparing CT with diagnostic laparoscopy in hidden peritoneal metastases detection. Among the 385 patients analyzed, diagnostic laparoscopy found hidden peritoneal metastases in 33 (8.5%). The main site was the greater omentum (38.6%), followed by the parietal and perihepatic peritoneum (22.8%)^24^. Ramos *et al.* (2016) carried out a systematic review with meta-analysis including 240 patients. The authors found that most patients were already in advanced stages, with mean resectability of only 68.7% after staging laparoscopy. The sensitivity was 84.6% while specificity was 100%. Hence, based on the results of this meta-analysis, we can conclude that staging laparoscopy for GC is a useful method for detecting peritoneal metastases, with good precision^32^. Currently, a Dutch multicenter prospective study is underway with the goal to compare PET-CT with staging laparoscopy in the preoperative resectable GC patients. The authors of this study hypothesize that there may be a 27% change in the treatment strategy, leading to an important cost reduction^11^. 

In the era of neoadjuvant chemotherapy (NAC), staging laparoscopy is most needed. Despite great advances in imaging methods, laparoscopy allows a thorough evaluation of the tumor, adjacent organs and the peritoneal cavity. According to a study by Bintintan *et al.* (2018), staging laparoscopy added important information in 65% of cases with NAC indication and changed the treatment strategy in 30% of them^8^.

#### Statement 8

Peritoneal washing with oncotic cytology should be performed in all cases during staging laparoscopy and / or surgery. It may be omitted if there is frank peritoneal carcinomatosis. 96% Agreement (level of evidence B; degree of recommendation 2a)

#### Comment

Some studies indicate an incidence between 7% and 10% of positive cytology in patients with GC without peritoneal metastases. This situation undoubtedly influences the type of treatment and the prognosis of these patients. In the meantime, peritoneal cytology may be omitted in patients whose lesions have a low risk for peritoneal dissemination (T1/T2, N0). It can also be avoided in those with clear peritoneal disease, in which a biopsy must be collected instead. Conventional peritoneal washing has low sensitivity and immunohistochemical and molecular techniques may be used to increase detection. Negativity cytology must be interpreted according to patient`s disease context and the surgeon`s impression^39^.

#### Statement 9

Analysis of serum tumor markers (CA19.9, CEA, CA 72.4) should be performed in all cases of gastric cancer. 56% Agreement (level of evidence B; degree of recommendation 2a)

#### Comment

Several tumor markers have been described in GC treatment, with different degrees of clinical applicability. Among them are the carcinoembryonic antigen (CEA), carbohydrate antigen 19.9 (CA 19.9), CA 72.4, CA 50, sialyl Tn antigen and alphafeto protein. A meta-analysis published in 2014 showed that the 3 markers most used in clinical practice are CEA, CA 19.9 and CA 72.4, with a sensitivity at the time of diagnosis of 24%, 27% and 30%, respectively^34^. Takahashi *et al.* (2003) demonstrated that the sensitivity and specificity for recurrence of CEA was 65.8% and 81.8% and for CA 19.9 was 55.0% and 93.7%, respectively. If these markers were elevated prior to surgery, the sensitivity of each marker was greater than 90%^37^. Similar results were found by Marrelli *et al.* (2001)*,* in which the sensitivity for recurrence of CEA was 44%, for CA19.9 was 56% and for CA 72.4 was 51%. The combined sensitivity of the 3 markers was 87%. It is interesting to mention that the increase in these markers preceded the clinical diagnosis in most cases. For patients with elevated tumor markers preoperatively, sensitivity was 100%. On the other hand, false elevations of CEA, CA 19.9 and CA 72.4 in disease-free patients were 21%, 26% and 3%, respectively^26^. Thus, the analysis of tumor markers should be performed in a combined manner, but only the CA 72.4 positivity should be considered as a specific indicator for GC recurrence during follow-up.

### Preoperative care statements

#### Statement 11

Multidisciplinary therapeutic planning (surgeon, endoscopist, general clinician, oncologist, radiologist and pathologist) is recommended before starting any type of treatment. 90% Agreement (level of evidence B; degree of recommendation 1)

#### Comment

Although there are guidelines directing the therapeutic approach in different stages, the individualities of each patient demand a personalized treatment, adapting the treatment to the patients and tumors features. The association of therapeutic modalities, and the choice of the strategy to be used, require the involvement of several specialists. Therapeutic planning in the form of a multidisciplinary tumor board alters the diagnosis formulated, and the treatment planned when compared to a single physician in 18 to 27%, and in 23 to 41%, respectively. There are no randomized studies, yet there are prospective studies and systematic review acknowledging the contribution of multidisciplinary planning in the therapeutic decision of patients with gastric GC^21^ ^,^ ^38^.

#### Statement 12

Patients who had weight lost greater than 10% of their usual weight in the past 6 months should receive some form of nutritional therapy before starting any treatment. 100% Agreement (level of evidence A; degree of recommendation 1) 

#### Comment

The cachexia in cancer patients is often associated with decreased intake, catabolism, and exacerbated inflammatory response. It affects among 50-80% of patients, accounting for about 20% of deaths. Even with limitations of use as an isolated parameter to assess nutritional status, weight loss greater than 10% in 6 months is indicative of severe malnutrition. In patients with moderate and severe malnutrition, nutritional input is required before any type of treatment, for 7-14 days, in order to reduce morbidity and mortality. Patients initially submitted only to neoadjuvant treatment, also have indication of nutritional therapy, particularly those with food intake below 70% of estimated energy spent^*13*^ ^,^ ^*46*^.

### Staging statements

#### Statement 10

Currently, the staging that must be adopted is the UICC/AJCC TNM 8^th^ edition. 100% Agreement (level of evidence A; degree of recommendation 2a)

#### Comment

The gastric cancer staging system, mainly with regard to the evaluation of the lymph node involvement (N), has undergone numerous changes in the last 20 years. The adoption of the numerical, quantitative system is due to the fact that it is simpler to be adopted in different centers of the world and has high precision. The last update of TNM - UICC / AJCC, 8th. edition, is consistent with the Japanese Gastric Cancer Association staging system, widely used in the East, and corrects the imperfections and disparities in the survival curves of stages IIIB and IIIC that existed in the 7th edition, since there was no separation between lymph node involvement between 7 and 15 and with more than 15 lymph nodes^33^. The Table 2 represents the TNM 8^th^ edition final staging. 

**TABLE 2:** TNM final staging (8^th^ Edition)

	N0	N1	N2	N3a	N3b
T1	IA	IB	IIA	IIB	IIIB
T2	IB	IIA	IIB	IIIA	IIIB
T3	IIA	IIB	IIIA	IIIB	IIIC
T4a	IIB	IIIA	IIIA	IIIB	IIIC
T4b	IIIA	IIIB	IIIB	IIIC	IIIC

#### Statement 18

The UICC / AJCC recommends a minimum of 15 harvested lymph nodes to allow correct staging. 92% Agreement (level of evidence B; degree of recommendation 2b)

#### Comment

The AJCC / UICC classification recommends at least 16 LN for correct lymph node staging analysis. In fact, some studies have shown the potential benefit of extended lymphadenectomy in this regard. Verlato *et al.* (2009) found that limited lymphadenectomy (less than 16 LN removed) is associated with inadequate staging in 54.4% of patients. On the contrary, these rates decreased to 6.2% and 1.4%, respectively, in patients undergoing more extensive lymphadenectomy (D2 or D3), which are associated with adequate disease staging in most cases^40^. 

#### Statement 19

D2 lymphadenectomy recommends at least 25 harvested lymph nodes. 76% Agreement (level of evidence B; degree of recommendation 2b)

#### Comment

The lymphadenectomy does not concern the number of lymph nodes, but the locations (stations) dissected. This methodology for guiding the removal of lymph node stations is based on results of lymph node involvement studies in various types of tumor (location, degree of tumor penetration into the gastric wall and histological type), associating it with the survival observed according to the dissection pattern. Respecting this standardization, it is unlikely that the number of LN removed in D2 lymphadenectomy will be less than 25. In 2011, a new Japanese guideline was published to coincide with the standard AJCC/UICC TNM classification. The type of lymphadenectomy started to be considered depending on the type of gastrectomy to be performed (total or subtotal)^3^.

#### Statement 20

It is recommended at the end of each operation that a member of the surgical team send the surgical specimen for the pathological analysis with all the separate and identified lymph node stations. 90% Agreement (level of evidence C; degree of recommendation 2b)

#### Comment

The wrong analysis of the number of lymph nodes removed after GC surgery can impair the correct staging for the presence of lymph node metastasis. This would certainly have a significant impact on the prognostic assessment and strategic formulation of adjuvant therapy. Under the premise of standard D2 lymphadenectomy, the number of lymph nodes collected depends mainly on the procedures that will examine the lymph nodes. Despite the fact that the current staging system consider only the number of lymph nodes involved, sending to the pathological evaluation the whole specimen, without dissection of the lymph node stations, could difficult this analysis, and furthermore, diminishes the quality of the surgery itself^42^.

### Endoscopic treatment statements

#### Statement 13

Endoscopic resection is indicated in well-differentiated adenocarcinoma tumors, restricted to the mucosa (T1a), less than 2 cm in its longest axis and not ulcerated. 100% Agreement (level of evidence B; degree of recommendation 1)

#### Comment

These are tumors whose recommendation for endoscopic treatment is supported by the main medical societies involved in the treatment of early GC, including the International Gastric Cancer Association, due to low risk of lymph node metastasis, and high survival rates, similar to those achieved by standard gastrectomy. It presents as a potential advantage over surgical treatment, due to the possibility of a less invasive curative approach. However, it requires accurate endoscopic and histological diagnosis, and experienced professionals to perform the procedure. Despite this recommendation is widely accepted, there are no randomized studies, and the current recommendations are based on non-randomized and retrospective studies^12^ ^,^ ^19^.

#### Statement 14

Early lesions with invasion of the submucosal layer, ulcerated, diffuse type and larger than 2 cm are exception criteria for endoscopic resection and should be adopted only in patients at high surgical risk. 92% Agreement (level of evidence B; degree of recommendation 2b)

#### Comment

The risk of lymph node metastases, incomplete resections, and recurrences make the above characteristics exception criteria for endoscopic resections. The endoscopic approach in these situations does not reproduce the results of radical surgical treatment, and should be reserved for peculiar situations, such as in patients who do not present performance for surgical treatment or refuse standard treatment. In the event of adoption of this approach, the need for exhaustive multidisciplinary discussion is emphasized, full clarification and agreement of the patient are mandatory, because the chance of complications, in such endoscopic procedure is not negligible, including perforations, which may result in possible surgical indication^2^ ^,^ ^29^ ^,^ ^30^.

#### Statement 15

Endoscopic submucosal dissection (ESD) is recommended as the treatment of choice for most superficial gastric tumors. 76% Agreement (level of evidence B; degree of recommendation 2a)

#### Comment

The submucosal dissections offer a higher probability of complete resections, excisions in a single fragment, obtaining free margins, and results in lower risk of recurrences than mucosal resections, especially in cases of tumors greater than 1cm. However, these are procedures of greater complexity than mucosal resections, and the risk of complications, especially perforations, is higher. They require high cost specialized instruments and the time necessary for performing such procedures is longer. Still, it represents a treatment modality with high curative potential, when formal clinical indications are observed (including cancer restricted to the mucosa non-ulcerated, regardless of tumor size, cancer restricted to the mucosa ulcerated maximum of 3 cm in diameter and cancer in the submucosal layer (<500 mm of the muscularis of the mucosa maximum of 3 cm in diameter non-ulcerated, all of the intestinal type and well differentiated.) In these situations, the possibility of lymph node metastases in early GC is practically nil^16^ ^,^ ^47^. 

### Follow-up statements

#### Statement 62

Patients with metastatic gastric cancer who have not responded to palliative chemotherapy or in poor clinical conditions, should receive only palliative care with best support of care. 96% Agreement (level of evidence C; degree of recommendation 1)

#### Comment

“Primum non nocere” (first, do not harm). In cases of patients with metastatic GC who do not respond to palliative chemotherapy or in poor clinical conditions, the goal of treatment is not to achieve a cure. It is essential to know when to stop. The medical assistant should seek to relieve symptoms, prevent complications and try to prolong life without impairing the quality of life^1^.

#### Statement 63

Patients undergoing radical surgery or after adjuvant therapy should not be followed due to the high cost and because there is no evidence that the follow-up improves survival. 18% Agreement (level of evidence C; degree of recommendation 3)

#### Comment

The periodic clinical follow-up of patients undergoing radical gastric surgery for GC is the subject of much controversy. That is because the treatment options for relapse historically have always been very limited and also because there are no randomized trials that confirm the usefulness of follow-up. In 2012, the Italian Study Group on Gastric Cancer held a 3-month debate entitled “Rationale and limits of oncological follow-up after gastrectomy for cancer”. This discussion involved 32 authors from 12 countries, including Brazil. Substantial differences emerged between the participants: authors from Japan, South Korea, Italy, Brazil, Germany and France perform routine follow-up with serial examinations, while authors from Eastern Europe, Peru and India do not. British and American surgeons carry out surveillance in a very limited way or in experimental studies^5^. In addition to the physical examination, performance status, weight, tomography, endoscopy and laboratory tests are used, including tumor markers are requested. With the progressive increase in clinical and surgical therapeutic options for recurrence, this topic has been recently discussed. A propensity-score match analysis showed that standardized follow-up significantly increased overall survival. This work also suggests that imaging tests and relapse treatment were associated with better outcomes. In addition, this study recommends CT scans periodically, especially in the first three years^35^. In fact, the possibility of recurrence is usually concentrated in the first 3 years in more than 90% of cases^9^. Other reasons for carrying out this postoperative follow-up are the possibility of diagnosing early and late gastrectomy complications; psychological support and surveillance of nutritional aspects of these patients.

#### Statement 64

Patients submitted to radical surgery can be followed through abdominal ultrasound, due to its accessibility and low cost. 38% Agreement (Level of Evidence C; degree of recommendation 3)

#### Comment

No evidence is available comparing imaging modalities performance in the diagnosis of recurrence during follow-up though. Nonetheless, the lack of efficacy regarding abdominal ultrasound in diagnosing some of the most common relapse sites of GC, such as peritoneal, regional and distant lymph nodes, or even small hepatic nodules, should be taken under consideration. Therefore, abdominal ultrasound should not be recommended routinely for GC follow-up^4^. 

#### Statement 65

In the postoperative period of patients submitted to radical surgery, the upper endoscopy is indicated when there is clinical suspicion of recurrence and digestive symptoms. 78% Agreement (level of evidence C; degree of recommendation 2a)

#### Comment

The local recurrence in GC patients treated with adequate resection margins and lymph node dissection is not common. It is reported in up to 10% of patients from multicentric trials, such as the Dutch Trial^36^. In the long term, among patients who had a subtotal gastrectomy, a recent Japanese study identified 5% risk of new tumors in the gastric stump^18^. Although these are not very high numbers, they justify the performance of upper endoscopy as a strategy for GC patients follow-up. Two questions remain though: the time interval between each exam and its use among patients who underwent a total gastrectomy. There is no conclusive evidence on these questions. The most recent guidelines recommend and upper endoscopy one year after surgery and every two years after that. Regarding the use of upper endoscopy after a total gastrectomy, it could be implied that it would be more relevant for patients with tumors located in the cardia/fundus^2^ ^,^ ^41^. 

#### Statement 66

The long-term follow-up should be offered to patients undergoing radical surgery or after the end of adjuvant therapy for nutritional and psychological control and support, early detection of recurrence, treatment of complications and data collection. 100% Agreement (level of evidence B; degree of recommendation 2b)

#### Comment

The main reason for monitoring patients operated for GC is the early diagnosis of recurrence, which usually occurs in the first 2 years after surgery with a curative intention. Of these patients, about 2/3 will have recurrence during follow-up. Despite the limited potential for treating recurrence, even when diagnosed early, other aspects influence the periodic surveillance of these patients. Among them, the treatment of complications related to gastrectomy, nutritional support, psychological support, diagnosis of other possible tumors, improvement in quality of life, data collection for institutional assessment of treatment outcomes^23^ (see statement 63 for more information).

#### Statement 67

The attempt of surgical resection in patients with single local recurrence and low surgical risk can be considered in selected cases. 98% Agreement (level of evidence C; degree of recommendation 3)

#### Comment

The laparotomy with curative intent is an option for those patients with inadequate resections (for instance: positive gastric margin, immediate postoperative with gross macroscopic nodal disease, D0/D1 lymphadenectomy and local lymph node relapse). Few reports exist showing well succeed resection of local or liver recurrence. Mostly more disease than anticipated is found or resection is abandoned due to safety issues. The procedure should be reserved for patients with good clinical status^28^.

## DISCUSSION

The statements conception that forged the II Brazilian Consensus on Gastric Cancer by ABCG was the subject of great debate (in the good sense of the word) among its authors. This is because some of the authors considered that the consensus should be succinct and concise and, therefore, with fewer statements. However, the understanding and perception of all forms of approach and management of GC require wide discussion. As previously mentioned extensively, the disease is complex and with different forms of behavior. In addition to this characteristic, is the fact that there is great social inequality in our country, causing enormous variability in the availability of human, diagnostic and technological resources between the different regions. For this reason, the simple adoption of foreign guidelines does not correspond to the needs found, requiring the positioning of ABCG for the best practices in our scenario.

Hence, it was decided to build up the II Consensus composed of sufficient statements that could cover the different aspects of GC as fully as possible. However, publishing only the results could raise doubts about how would be the best way to manage a patient with GC according to each local reality. In fact, among the 67 statements performed, there was 100% agreement in just 10 (15%). Some aspects are still cause for much discussion among specialists, such as the routine use of drains in GC surgery, the role of neoadjuvant chemotherapy, the best form of surgical approach in cardia tumors, among others. Thus, the ABCG’s main goal was to provide arguments based on the literature that could somehow support, or not, the guidelines contained in each statement. This will allow each professional to, as far as possible, offer adequate and effective treatment to the patient with GC.

On the other hand, the literature review carried out on each statement made the II Consensus somewhat extensive. Therefore, it became opportune to publish the Brazilian Gastric Cancer Guidelines in two stages. In this first part are the statements regarding the diagnosis, staging, endoscopic treatment and follow-up. In the next stage (part 2) will be presented the statements about the treatment itself (surgical and multimodal).

## CONCLUSION

This publication contains comments and guidelines with reference to the diagnosis, staging, endoscopic treatment and follow-up of GC patients found on the II Brazilian Consensus on Gastric Cancer. The guidelines shown here are intended to assist professionals working in the fight against GC with relevant and current information, allowing them to be applied in daily medical practice.
